# Differential Distribution of Ca^2+^ Channel Subtypes at Retinofugal Synapses

**DOI:** 10.1523/ENEURO.0293-20.2020

**Published:** 2020-11-04

**Authors:** Gubbi Govindaiah, Peter W. Campbell, William Guido

**Affiliations:** Department of Anatomical Sciences and Neurobiology, University of Louisville, School of Medicine, Louisville, KY 40292

**Keywords:** dorsal lateral geniculate nucleus, N-type Ca^2+^ channel, P/Q-type Ca^2+^ channel, retinal synapse, superior colliculus

## Abstract

Retinofugal synapses serve as models for understanding how sensory signals from the periphery are relayed to the brain. Past studies have focused primarily on understanding the postsynaptic glutamatergic receptor subtypes involved in signal transmission, but the mechanisms underlying glutamate release at presynaptic retinal terminals remains largely unknown. Here we explored how different calcium (Ca^2+^) channel subtypes regulate glutamatergic excitatory synaptic transmission in two principal retinorecipient targets, the dorsal lateral geniculate nucleus (dLGN) and superior colliculus (SC) of the mouse. We used an *in vitro* slice preparation to record the synaptic responses of dLGN and SC neurons evoked by the electrical stimulation of optic tract (OT) fibers before and during the application of selective Ca^2+^ channel blockers. We found that synaptic responses to paired or repetitive OT stimulation were highly sensitive to extracellular levels of Ca^2+^ and to selective antagonists of voltage gated Ca^2+^ channels, indicating that these channels regulate the presynaptic release of glutamate at retinal synapses in both dLGN and SC. Bath application of selective Ca^2+^ channel blockers revealed that P/Q-type Ca^2+^ channels primarily operate to regulate glutamate release at retinal synapses in dLGN, while N-type Ca^2+^ channels dominate release in the SC.

## Significance Statement

The retinofugal synapse serves as the preeminent model for understanding how sensory information from the external world is relayed to the brain. We explored the cellular mechanisms that regulate the presynaptic release of glutamate in two principal retinorecipient targets, the dorsal lateral geniculate nucleus (dLGN) and superior colliculus (SC) of mouse. Although the great majority of retinal ganglion cells (RGCs) have an axon that bifurcates and projects to both dLGN and SC, different calcium (Ca^2+^) channel subtypes regulate the presynaptic release of glutamate, with P/Q channels largely operating in dLGN, and N-type in SC. Because these subtypes possess a unique set of biophysical properties that can affect the efficacy of synaptic transmission, such nonuniform distribution promotes terminal specific modulation of neurotransmitter release.

## Introduction

Visual information from the eye is conveyed to the brain by retinal ganglion cells (RGCs). While RGC axons project to a number of subcortical structures, almost all of them terminate in the dorsal lateral geniculate nucleus of thalamus (dLGN) and superior colliculus (SC) of the midbrain (Kerschensteiner and Guido, 2017). Signal transmission through these structures play important roles in the relay of visual signaling that gives rise to the conscious perception of images (dLGN), visually guided behavior (SC), as well as reflexive eye and head movement (SC). Indeed, retinofugal synapses in these structures have been the subject of intense investigation and serve as models for understanding how sensory signals from the periphery are relayed to the brain (Isa and Hall, 2009; Sherman and Guillery, 2011; Cang et al., 2018; Guido, 2018; Ito and Feldheim, 2018). To date, studies have focused on elucidating the postsynaptic glutamatergic receptor subtypes, and how their ligand-gated kinetics affect the speed, duration, intensity, as well as the plasticity of signal transmission (Salt, 2002; Liu and Chen, 2008; Furman and Crair, 2012; Furman et al., 2013; Hauser et al., 2013, 2014; Litvina and Chen, 2017). However, the mechanisms underlying glutamate release at presynaptic retinal terminals in these subcortical structures remain poorly understood.

Typically, at central excitatory synapses presynaptic glutamate release is regulated by at least three voltage activated calcium (Ca^2+^) channel subtypes, N-type, P/Q-type, and R-type (Catterall, 2000; Reid et al., 2003; Kamp et al., 2012). While the Ca^2+^ influx associated with their activation triggers vesicle fusion and neurotransmitter exocytosis, each of these channel subtypes possess a unique set of biophysical properties that can affect the efficiency of transmitter release, as well as influence the degree and polarity of short-term, use-dependent synaptic plasticity (Zucker and Regehr, 2002; Catterall and Few, 2008; Fioravante and Regehr, 2011). Thus, knowledge about Ca^2+^ channel subtype distribution and function is important if we are to understand retinofugal transmission.

Here, we explored whether different subtypes of voltage activated Ca^2+^ channels regulate glutamatergic excitatory synaptic transmission in two primary retinorecipient targets, the dLGN and SC of the mouse. We used an *in vitro* slice preparation to conduct whole cell recordings of the synaptic responses of dLGN and SC neurons evoked by the electrical stimulation of optic tract (OT) fibers before and during the application of selective Ca^2+^ channel subtype blockers. Although the great majority of RGCs have axons that bifurcate and project to both dLGN and SC (Dhande et al., 2011; Ellis et al., 2016), different Ca^2+^ channel subtypes regulate the presynaptic release of glutamate, with P/Q-type channels largely mediating synaptic transmission in dLGN, and N-type in SC.

## Materials and Methods

All experimental procedures were reviewed and approved by the author’s institutional animal care and use committee and conducted in accordance with the Society for Neuroscience policies on the use of animals in research. Experiments involved 26 adult C57/BL6 mice (postnatal days 40–60) of either sex.

Acutely prepared brain slices and *in vitro* whole cell recordings were made using conventional methods (Dilger et al., 2011, 2015; Bickford et al., 2015; Charalambakis et al., 2019). Coronal slices at the level of dLGN or sagittal slices at the level of SC were cut (280 μm thick) on a vibratome, incubated in oxygenated (95% O_2_/5% CO_2_) artificial CSF (ACSF) containing the following: 126 mm NaCl, 26 mm NaHCO_3_, 2.5 mm KCl, 1.25 mm NaH_2_PO_4_, 2 mm MgCl_2_, 2 mm CaCl_2_, and 10 mm glucose at 32°C for 30 min, and later maintained at room temperature (22–24°C). For some experiments, the external Ca^2+^ (Ca_e_) concentration was adjusted from 2.0 mm to 1.5 and 3.0 mm.

Visualized, whole-cell patch recordings were obtained from dLGN and SC neurons. Borosilicate glass pipettes were pulled from vertical puller (Narishige) and had a tip resistance of 5–10 MΩ when filled with an internal solution containing the following:117 mm K-gluconate, 13.0 mm KCl, 1 mm MgCl_2_, 0.07 mm CaCl_2_, 0.1 mm EGTA, 10 mm HEPES, 2 mm Na-ATP, and 0.4 mm Na-GTP. Biocytin (0.5%) was also included in the internal solution to allow for intracellular filling and subsequent reconstruction using confocal microscopy (Charalambakis et al., 2019). The pH and osmolality of internal solution were adjusted to 7.3 and 290 mOsm, respectively. Brain slices were transferred to a recording chamber that was maintained at 32°C and continuously perfused with ACSF (3.0 ml/min). Neurons were visualized using an upright microscope (BX51W1, Olympus) equipped with differential interference contrast optics. Whole-cell recordings were obtained using a Multiclamp 700B amplifier (Molecular Devices), signals were sampled at 2.5–5 kHz, low-pass filtered at 10 kHz using a Digidata 1320 digitizer and stored on computer for subsequent analyses using pClamp software (Molecular Devices). Access resistance (<15 MΩ) was monitored continuously throughout the experiment, and neurons in which access resistance changed by >20% were discarded. A 10-mV junction potential was subtracted for all voltage recordings.

To evoke synaptic activity, square-wave pulses (0.1–0.3 mV, 25–200 μA) were delivered at variable rates (0.5–20 Hz) through a pair of thin gauged tungsten wires (0.5 MΩ) positioned in the OT near the targeted structure. EPSCs were evoked at a holding potential of –70 mV. In some experiments, synaptic responses were recorded in current clamp mode at resting membrane potentials (−60 to −76 mV).

All drugs were bath applied. To examine the role of Ca^2+^ channel subtypes underlying excitatory synaptic transmission, the following Ca^2+^ channel blockers were used: N-type, ω-conotoxin GVIA (ω-CgTx GVIA, 1 μm, Alomone Labs C-300) and PD173212 (Tocris Bioscience, 3552); P/Q-type, ω-agatoxin IVA (ω-Aga IVA, 0.2–0.4 μm, Alomone Labs, STA-500); L-type, nimodipine (10 μm, Tocris Bioscience, 0600); and R-type, SNX 482 (500 nm to 1 μm, Alomone Labs, RTS-500). Ca^2+^ channel blockers were prepared as concentrated stocks in distilled water, stored at −70°C and diluted to working concentrations just before use. Some stock solutions were prepared in dimethyl sulfoxide (DMSO). The final DMSO concentration in ACSF never exceeded 0.1% (v/v). To isolate ESPC activity and block IPSCs, the GABA_A_ receptor antagonist 4-[6-imino-3-(4-methoxyphenyl)pyridazin-1-yl]butanoic acid hydrobromide (SR95531, 10 μm, Tocris 1262) was applied. In some cases, experiments were performed in the presence of the NMDA receptor antagonist d(−)4-(3-phosphonopropyl)piperazine-2-carboxylic acid (d-CPP, 20 μm, Tocris 1265) and AMPA receptor antagonist 6,7-dinitroquinoxaline-2,3-dione (DNQX, 40 μm, Tocris 2312).

All off-line data analysis was done using pClamp 10 software (Molecular Devices). Predrug or control data were collected for at least 3–5 min before drug application and then 5–10 min thereafter. All measurements involved a maximal response to OT stimulation and were based on three to five stimulus presentations. The paired-pulse ratio (PPR) was determined by dividing the amplitude of the second EPSC by the amplitude of the first EPSC (EPSC_2_/EPSC_1_). For statistical analyses, Prism software (GraphPad) was used. Student’s *t* tests (paired and unpaired) were used for comparison as indicated, *p* < 0.05 was taken as significant. For estimates of effect size, Figures 2*B*, 3*C*, 4 provide individual data along with difference plots and 95% confidence intervals (95CIs).

## Results

*In vitro* whole-cell recordings from acutely prepared brain slices were obtained from a total of 52 dLGN and 50 SC neurons. For dLGN, we recorded from relay cells as defined by their electrophysiological properties and in many cases their dendritic morphology which was reconstructed from biocytin fills conducted during the recording (Krahe et al., 2011; El-Danaf et al., 2015). For SC, we targeted the neurons in the main retino-recipient region, stratum griseum superficialis (Furman and Crair, 2012; Gale and Murphy, 2014).

**Figure 1.**
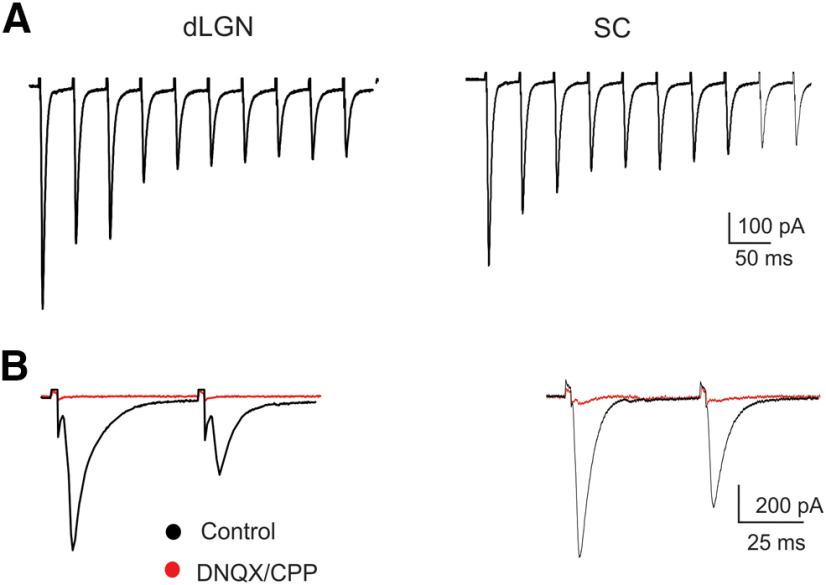
Excitatory glutamatergic synaptic transmission in dLGN and SC. ***A***, Whole-cell voltage-clamp recordings showing the excitatory postsynaptic activity evoked by repetitive electrical stimulation of the OT for dLGN (left) and SC (right) neuron. Repetitive stimulation at 20 Hz evoked a train of EPSCs that rapidly depressed with each successive pulse. ***B***, Paired stimulation (ISI, 50 ms) also led to strong paired-pulse depression in which the amplitude of the second (EPSC_2_) is greatly reduced compared with the initial one (EPSC_1_). Responses were abolished (red traces) by the bath application of glutamate receptor antagonists (AMPA: DNQX 25 μm) and (NMDA: d-CPP 20 μm). All responses recorded at −70 mv.

**Figure 2.**
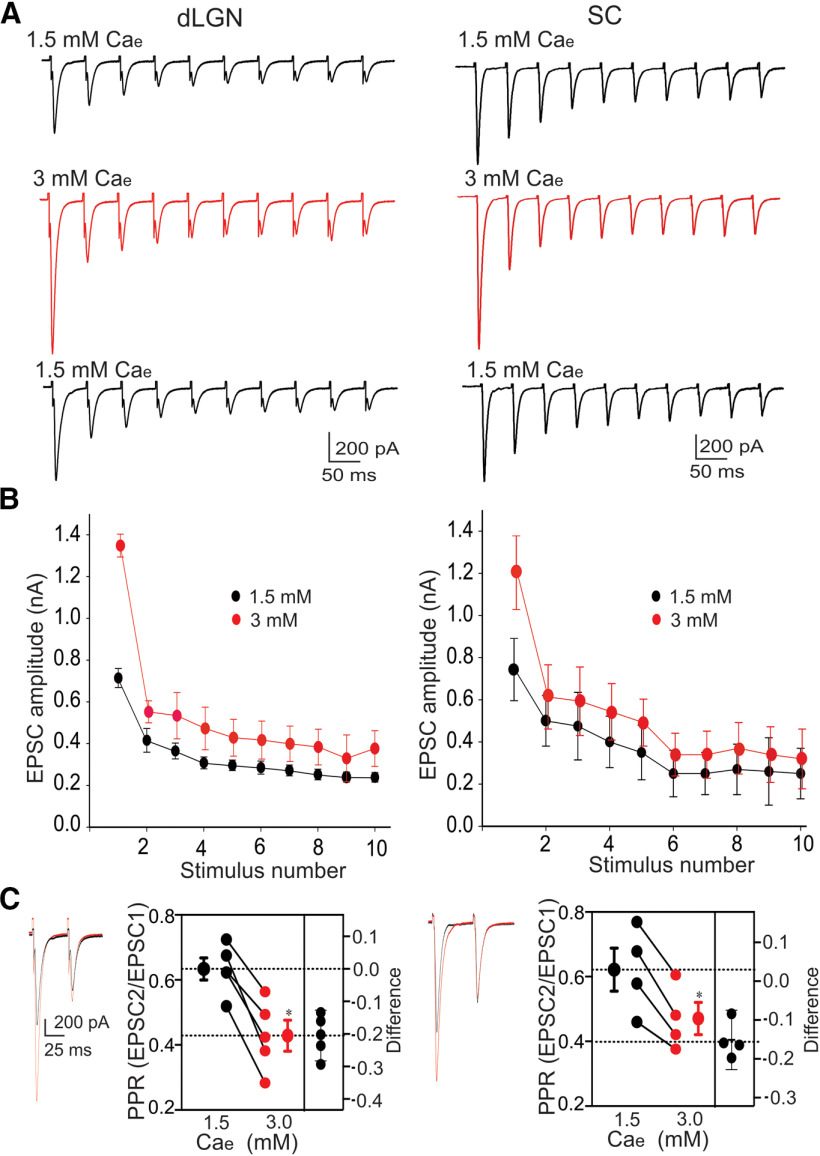
The effects of Ca_e_ on dLGN and SC synaptic responses. Whole-cell voltage-clamp recordings showing the EPSCs evoked by repetitive OT stimulation. ***A***, Examples of dLGN (left) and SC (right) EPSCs evoked by repetitive stimulation at 20 Hz during wash-in of 1.5 mm Ca_e_, followed by 3.0 mm and then 1.5 mm Ca_e_. ***B***, Below each example are the summary plots for dLGN (*n *=* *6) and SC (*n *=* *6) neurons showing mean ± SD changes in EPSC amplitude as a function of stimulus number within the stimulus train during 1.5 Ca_e_ (black symbols) and 3.0 mm Ca_e_ (red symbols) ***C***, Examples of dLGN (left) and SC (right) EPSCs evoked by paired OT stimulation (50-ms ISI) during wash-in of 1.5 mm Ca_e_ (black) and 3.0 mm Ca_e_ (red). Adjacent summary plots depict PPRs (left *y*-axis) for individual neurons (dLGN, *n* = 5; SC, *n* = 4) as well as group mean ± SEM values (large symbols). Also included (right *y*-axis) are paired differences for each neuron, along with error bars that reflect the 95CI. Dotted horizontal lines depict the group mean at 1.5 mm Ca_e_ (top) and the average difference between group means (effect size). For both dLGN and SC neurons, an increase in Ca_e_ led to larger initial responses and stronger paired pulse depression (dLGN *p =* *0.0019; SC *p =* *0.0081). All responses recorded at −70 mv.

As expected, for both dLGN and SC neurons, electrical stimulation of the OT evoked large EPSCs (Fig. 1*A*,*B*) that exhibited the hallmark features of driver-like or Class 1 responses (Sherman and Guillery, 2011; Sherman, 2012). Bath application of ionotropic glutamate antagonists (DNQX 40 μm, D-CPP 20 μm) abolished synaptic activity (dLGN, *n *=* *4; SC, *n *=* *5; Fig. 1*B*), indicating that retinally evoked EPSCs were mediated by AMPA and NMDA receptor activation. Repetitive stimulation at 20 Hz [50-ms interstimulus intervals (ISIs)] also evoked a train of EPSCs that decreased in amplitude with each successive stimulus pulse (Fig. 1*A*,*B*). To quantify the degree of synaptic depression, we generated PPRs in which the amplitude of the initial response was compared with the second one (EPSC_2_/EPSC_1_; Figs. 1*B*, 2*C*, 4). Both dLGN and SC neurons showed strong paired pulse depression. The average PPR for dLGN neurons was 0.48 ± 0.08 (*n *=* *6) and for SC neurons, 0.52 ± 0.09 (*n = *6), indicating that the amplitude of the initial EPSC was ∼1.4- to 1.7-fold larger than the second one (Fig. 1*B*). The decreases in synaptic strength associated with PPRs likely reflect the depletion in the presynaptic terminal readily releasable pool of glutamate-containing vesicles (Zucker and Regehr, 2002; Chen and Regehr, 2003; Catterall and Few, 2008; Fioravante and Regehr, 2011; Regehr, 2012).

Postsynaptic responses in dLGN and SC were also regulated by the concentration of extracellular Ca^2+^ (Ca_e_; Fig. 2). An increase in the Ca_e_ from 1.5 to 3.0 mm led to a ∼2-fold increase in the initial EPSC amplitude that was evoked by trains or paired OT stimulation. In both Ca_e_ conditions, EPSCs continued to decline in amplitude with each successive pulse in the stimulus train before eventually reaching a steady state (Fig. 2*A*). However, PPRs were significantly decreased in neurons (dLGN 32%; SC 24%) where Ca_e_ was altered from 1.5 to 3.0 mm (dLGN, *n *=* *5, mean ± SEM, 0.63 ± 0.03 vs 0.42 ± 0.04, *t* test *p* = 0.0019, effect size = −0.21, 95CI = −0.28, −0.13; SC, *n *=* *4, 0.62 ± 0.07 vs 0.47 ± 0.05, *t* test *p *=* *0.0081, effect size = −0.15, 95CI = −0.23,−0.07; Fig. 2*C*). These decreases in synaptic strength prevail under conditions such as high Ca_e_, an event that promotes a high initial probability of transmitter release (Regehr, 2012; Thanawala and Regehr, 2013).

**Figure 3.**
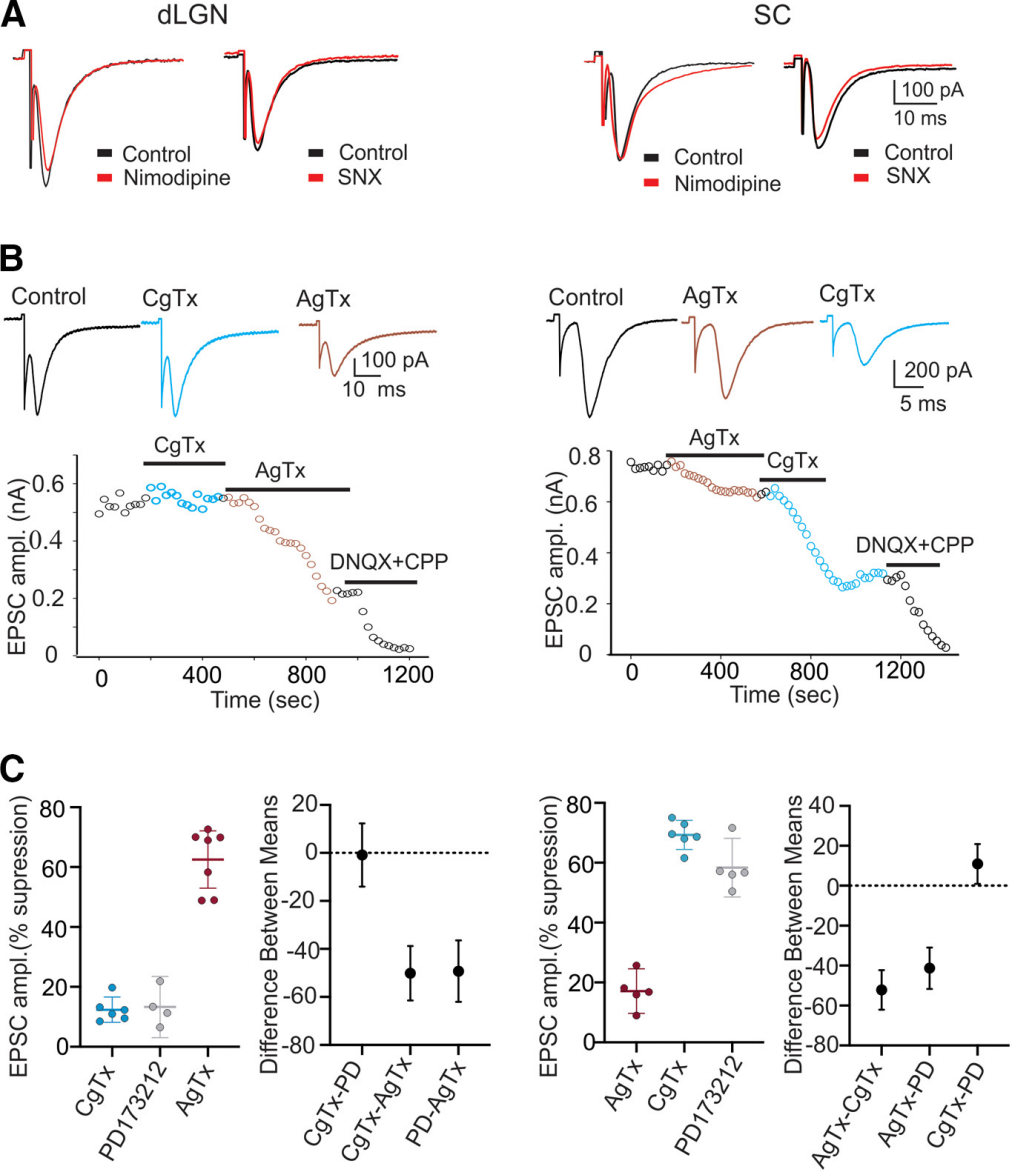
The effects of selective Ca^2+^ channel subtype blockade on the synaptic responses of dLGN and SC neurons. ***A***, Examples of EPSCs evoked by OT stimulation for dLGN (left) and SC (right) neurons before (black, Control) and during bath application of L-type channel blockade by nimodipine (red) and R-type channel blockade by SNX (red) L-type and R-type blockade had no effect on the synaptic responses of dLGN and SC neurons. ***B***, Examples of EPSCs evoked by OT stimulation for a dLGN and SC neuron before (Control, black) and during N-type blockade by ω-CgTx GVIA (CgTX, blue) and P/Q blockade by ω-Aga IVA (AgTX, red). Below each example are plots for an dLGN and SC neuron showing the changes in EPSC amplitude as a function of time before and during N (CgTX) and P/Q (AgTX) channel, and glutamate receptor (DNQX+CPP) blockade. The bar under each drug represents the time course and duration of drug application. ***C***, Summary plots for dLGN (*n* = 17) and SC neurons (*n* = 16) showing the degree of EPSC suppression associated with N-type blockade by ω-CgTx GVIA (blue) and PD173212 (gray), and P/Q blockade by ω-Aga IVA (red). Each point represents an individual neuron, with bars representing group means and SEMs. Adjacent graphs (right) depict differences between the means for each drug treatment. Symbols reflect difference means and error bars the 95CIs. Dotted horizontal line shows a value of 0 (no difference). N and P/Q channel blockade differentially regulated synaptic transmission. For dLGN, P/Q blockade by ω-Aga IVA (*n* = 7) led to a ∼65% reduction in EPSC, whereas N-type blockade by ω-CgTx GVIA (*n* = 6) or PD 173212 (*n* = 4) led to a 12–13% reduction. Differences between the means for these drug treatments showed that for dLGN, P/Q blockade reduced amplitude 50% more than N-type blockade. For SC neurons, N-type blockade by ω-CgTx GVIA (*n* = 6; 69%) or PD 173212 (*n* = 5; 58%) led to a 58–69% reduction in amplitude, whereas P/Q blockade by ω-Aga IVA (*n* = 5) led to a 17% reduction. Differences between the means showed that for SC neurons N-type blockade reduced amplitude between 41% and 52% more than P/Q blockade. All responses recorded at −70 mv.

To examine the role of different Ca^2+^ channel subtypes involved in retinofugal transmission, we measured the synaptic activity of dLGN and SC neurons evoked by OT stimulation, before and after bath application of specific Ca^2+^ channel blockers (Fig. 3). For dLGN and SC neurons, bath application of L-type Ca^2+^ channel blocker nimodipine (10 μm) had no effect on the amplitude of EPSCs evoked by OT stimulation (mean ± SEM; dLGN, *n *=* *8, predrug 641 ± 42.5 pA vs nimodipine 625 ± 38.6 pA, *t* test *p* > 0.6; SC, *n *=* *5, predrug 591 ± 43.5 pA, vs nimodipine 589 ± 50.2 pA, *t* test *p* > 0.5; Fig. 3*A*). These results are consistent with previous immunocytochemical reports showing an absence of labeling for L-type Ca^2+^ channels on retinal axon terminals but the presence of heavy labeling on somata and proximal dendrites of dLGN and SC neurons (Cork et al., 2001; Jaubert-Miazza et al., 2005; Dilger et al., 2011). When these channels are activated by excitatory postsynaptic activity, it leads to plateau-like depolarizations in dLGN and SC neurons (Lo and Mize, 2000; Lo et al., 2002; Dilger et al., 2011, 2015). Additionally, we found that selective blockade of R-type channels by bath application of SNX 482 (500 nm to 1 μm) had no effect on the synaptic responses of dLGN or SC neurons (mean ± SEM; dLGN, *n *=* *6, predrug 715 ± 53.8 pA vs SNX 482, 717 ± 47.3 pA, *t* test *p* > 0.1; SC, *n *=* *7, predrug 574 ± 53.8 pA vs SNX 482 556 ± 38.4 pA, *t* test *p* > 0.6; Fig. 3*A*). However, the application of specific blockers for P/Q and N-type channels (Fig. 3*B*,*C*) revealed these subtypes play a substantial, albeit differential role in regulating synaptic transmission in dLGN and SC. In dLGN, bath application of the N-type channel blocker ω-CgTx GVIA (1 μm) had little effect on EPSC amplitude, but the subsequent application of the P/Q-type blocker ω-Aga IVA (0.2–0.4 μm) led to a substantial reduction. By contrast, in SC, EPSC amplitude was greatly affected by N-type but not by P/Q-type blockade. Representative examples of these effects are shown in Figure 3*B*. For the dLGN neuron (Fig. 3*B*, left), EPSC amplitude remained relatively stable during a 5 min N-type channel blockade by ω-CgTx GVIA but showed an immediate and sustained reduction when P/Q channel blocker ω-Aga IVA was introduced. For the SC neuron (Fig. 3*B*, right), P/Q-type blocker ω-Aga IVA had a marginal effect on EPSC amplitude, but then showed a substantial attenuation during N-type blockade by ω-CgTx GVIA. In both instances, bath application of glutamate antagonists (DNQX+ CPP) eliminated remaining synaptic activity. Summary plots depicting the changes in EPSC amplitude and effect sizes between drug treatments during N-type channel blockade by ω-CgTx GVIA or PD173212 (5 μm), and P/Q blockade by ω-Aga IVA are shown in Figure 3*C*. For dLGN neurons, on average P/Q blockade by ω-Aga IVA (*n* = 7) led to a ∼65% reduction in EPSC amplitude compared with predrug baseline measures. By contrast, on average N-type blockade by ω-CgTx GVIA (*n* = 6) or PD173212 (*n* = 4) led to a 12–13% reduction in amplitude compared with baseline values. A comparison between these drug treatments (Fig. 3*C*, differences between means) revealed that for dLGN neurons P/Q blockade reduced amplitude 50% more than N-type blockade (ω-CgTx GVIA vs ω-Aga IVA effect size = −50.14, 95CI −61.48, −38.80; PD 173212 vs ω-Aga IVA effect size = −49.25, 95CI = −62.03, −36.47). For SC neurons, on average N-type blockade by ω-CgTx GVIA (*n* = 6; 69%) or PD 173212 (*n* = 5; 58%) led to a 58–69% reduction in amplitude compared with baseline values; whereas P/Q blockade by ω-Aga IVA (*n* = 5) only led to a 17% reduction. A comparison between these drug treatments (Fig. 3*C*, differences between means) showed that for SC neurons N-type blockade reduced amplitude between 41% and 52% more than P/Q blockade (ω-Aga IVA vs ω-CgTx GVIA effect size = −52.16, 95CI: −62.08, −42.25; ω-Aga IVA vs PD 173212 effect size = −41.26; 95%: −51.62, −30.90).

**Figure 4.**
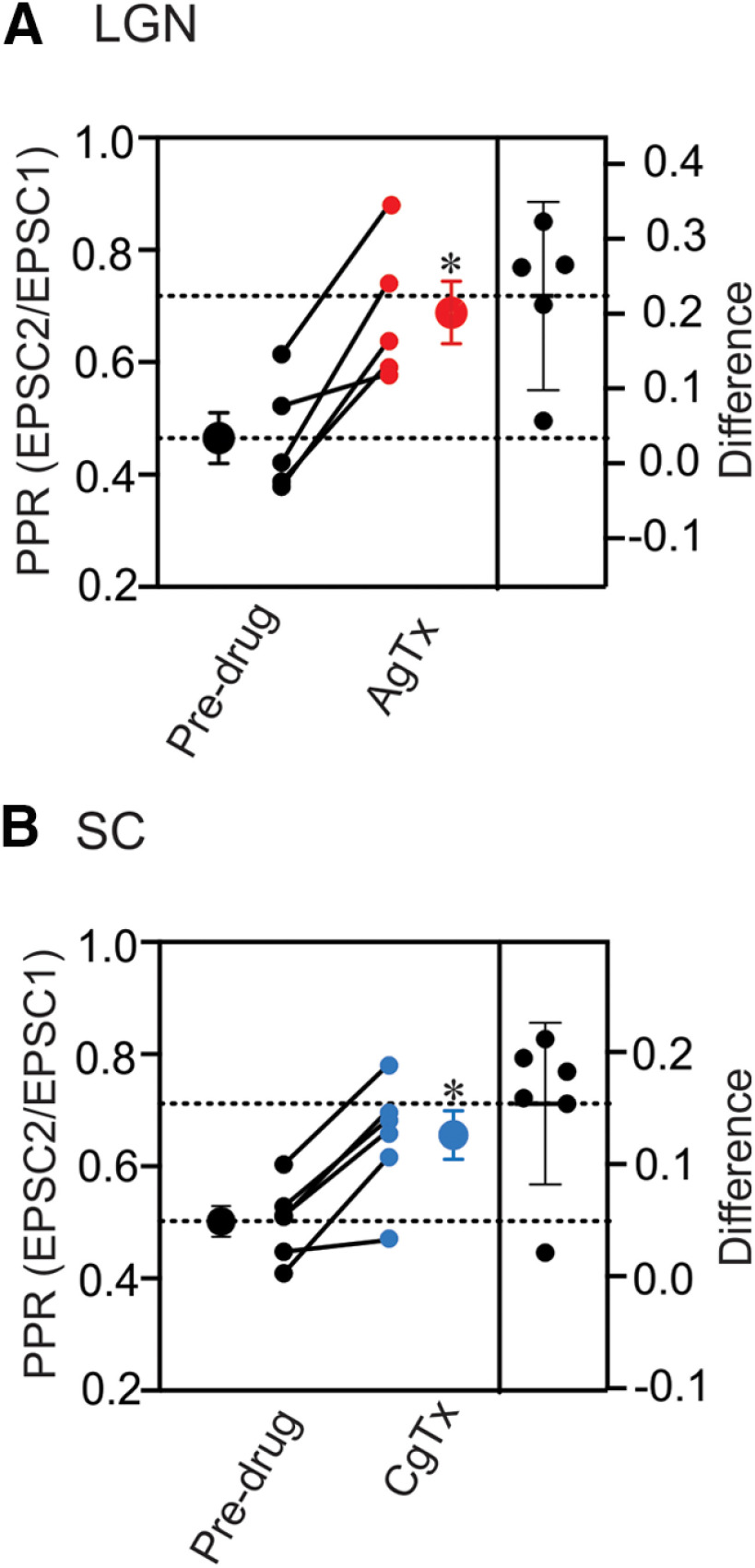
The effects of N and P/Q channel blockade on paired pulse depression in dLGN and SC neurons. ***A***, ***B***, Summary plots that depict PPRs for individual dLGN (***A***, *n* = 5) and SC (***B***, *n* = 6) neurons, along with the group mean ± SEM (large symbols). Also included (right *y*-axis) are paired differences for each neuron, along with error bars that reflect the 95CI. Dotted horizontal lines depict the group mean difference (effect size) during drug treatment (top) and the mean for predrug values. Selective blockade of P/Q for dLGN (*p =* *0.0078) and N for SC (*p =* *0.0027) led to an increase in PPRs, and thus a weakening in paired pulse depression. All responses recorded at −70 mv.

To further confirm that these changes in EPSC amplitude were presynaptic in nature we examined whether P/Q-type or N-type blockade altered PPRs of dLGN and SC neurons in response to paired OT stimulation (ISI, 50 ms). While the selective blockade of P/Q and N-type channels led to a 70–80% reduction in EPSC amplitude for dLGN and SC neurons, respectively, we tested whether the small excitatory currents that remained were subject to paired pulse depression. The PPRs for dLGN and SC neurons measured before and during Ca^2+^ channel blockade are shown in Figure 4. Similar to the effects associated with reduced levels of Ca_e_ (1.5 mm; Fig. 2)-selective blockade of these channels led to an increase in PPRs, thereby diminishing the degree of paired pulse depression (Fig. 4). For dLGN neurons (Fig. 4*A*), PPRs increased significantly following P/Q blockade by ω-AgTx IVA (*n* = 5, predrug 0.46 ± 0.05 vs ω-Aga IVA 0.69 ± 0.06, *t* test *p *=* *0.0078, effect size = 0.22, 95CI = 0.10, 0.35). For SC neurons, PPR increased significantly following N-type channel blockade by ω-CgTx GVIA (*n *=* *6, predrug 0.50 ± 0.03 vs ω-CgTx GVIA 0.66 ± 0.04, *t* test *p *=* *0.0027, effect size = 0.15, 95CI = 0.08, 0.23).

## Discussion

Here, we identified the Ca^2+^ channel subtypes that mediate synaptic transmission at two primary retinorecipient targets, the dLGN and SC. We found that blockade of P/Q channels greatly attenuated retinally-evoked EPSCs in dLGN, while the blockade of N-type reduced this excitatory activity in SC. Selective blockade of these Ca^2+^ channel subtypes also diminished the degree of paired pulse depression on the remaining synaptic activity, indicating that these pharmacological effects are likely presynaptic in origin (Zucker and Regehr, 2002; Catterall and Few, 2008; Fioravante and Regehr, 2011; Regehr, 2012). Taken together, these results suggest a differential gating of presynaptic glutamate release, with P/Q channels operating primarily at retinogeniculate synapses, and N-type at retinotectal ones. Both P/Q and N-type channels have been implicated in fast vesicle fusion and transmitter release and are often paired together at central excitatory synapses (Reid et al., 2003; Kamp et al., 2012). Indeed, immunocytochemical studies show that the pore forming subunit for N-type and P/Q channels are expressed in somata and along axons of RGCs (Sargoy et al., 2014). However, the degree of co-expression or localization within RGC terminals remains unknown. Nonetheless, their nonuniform distribution among presynaptic terminals, while rare, is not without precedence even when they arise from the same axon (Poncer et al., 1997; Reid et al., 1997; Scanziani et al., 1998; Rozov et al., 2001; Yamamoto and Kobayashi, 2018). In the case of retinal axons, tracing studies reveal that ∼80% of all RGCs that project to dLGN also terminate in SC (Ellis et al., 2016). Thus, it is conceivable that the target specific differences in the distribution of presynaptic Ca^2+^ subtypes noted here occur within a single RGC. Unfortunately, our *in vitro* recordings did not allow us to test for this likelihood. Consequently, we cannot rule out the possibility that the small fraction of RGCs that project exclusively to dLGN or SC (Dhande et al., 2011; Ellis et al., 2016) contributed to these target specific differences.

Why then do retinal axons have such target specific differences? While P/Q and N subtypes share similar channel kinetics, a number of reports suggest they may play unique roles in synaptic transmission (Reid et al., 2003; Kamp et al., 2012; Stanley, 2015) and that the nonuniform distribution among retinogeniculate and tectogeniculate projections could enable terminal specific modulation of neurotransmitter release. For example, brief action potentials activate P/Q more efficiently than N-type (Currie and Fox, 2002). P/Q channels may also have slightly faster activation and deactivation times along with a shallower voltage dependency than N-type (Naranjo et al., 2015). Overall these characteristics would promote highly efficient Ca^2+^ influx during brief depolarizations, especially during periods of high frequency activation (Li et al., 2007; Naranjo et al., 2015). The preponderance of P/Q-type channels at retinogeniculate synapses could help explain the high temporal fidelity of dLGN responses to stationary or moving stimuli (Piscopo et al., 2013; Ellis et al., 2016). P/Q-type and N-type also display differences in the sensitivity to G protein and metabotropic receptor interactions (Zamponi and Snutch, 1998; Kamp et al., 2012), in the degree of structural coupling to vesicles and release machinery (Wu and Borst, 1999; Alvarez et al., 2008; Álvarez et al., 2013), and in their ability to evoke short-term activity dependent synaptic plasticity (Currie and Fox, 2002; Catterall et al., 2013; Ricoy and Frerking, 2014; Yamamoto and Kobayashi, 2018). How such features apply to retinogeniculate and retinotectal synapses remains an open question since in rodents, the structural composition of these terminals as well as their postsynaptic response profiles are similar. Both retinogeniculate and retinotectal terminals are large, with pale mitochondria and form glomerular-like synaptic arrangements with GABAergic neurons (Boka et al., 2006; Bickford et al., 2010; Hammer et al., 2014; Masterson et al., 2019). Functionally, these synapses display driver-like characteristics, exhibiting large ionotropic glutamate responses that respond with synaptic depression to repetitive stimulation (Chen et al., 2002; Chandrasekaran et al., 2007; Furman and Crair, 2012; Bickford et al., 2015). One notable difference between these terminals is in the nature of their postsynaptic targets, with far more GABAergic neuronal subtypes in SC than dLGN (Whyland et al., 2020). From an evolutionary perspective, SC is also a more primitive structure than dLGN, which would be consistent with the prevalence of N-type channels found in lower vertebrates (Reid et al., 2003). Perhaps future studies will provide further insight as to the functional implications for such heterogeneity at these retinofugal synapses.

## Synthesis

Reviewing Editor: Nicholas J. Priebe, University of Texas at Austin Decisions are customarily a result of the Reviewing Editor and the peer reviewers coming together and discussing their recommendations until a consensus is reached. When revisions are invited, a fact-based synthesis statement explaining their decision and outlining what is needed to prepare a revision will be listed below.

SYNTHESIS

Two reviewers evaluated the manuscript “Differential distribution of Ca2+ channel subtypes at retinofugal synapses” and both highlighted the novelty and importance of the work. Both reviewers found the experiments and analyses compelling. They both noted in review and in consultation, however, that the morphologies shown in figure 1 should be quantified, or potentially removed. Further they noted that the present manuscript does not yet demonstrate directly that a single axon uses different channels depending on target organ, so conclusions should be modified to reflect this open issue. The reviewers provide detailed suggestions which you should find useful in revising your manuscript.

-

REVIEWER 1

ADVANCES IN THE FIELD

This paper very nicely shows yet another deviation, at least in principle, from Dale’s Law - retinal ganglion cells, while using glutamate at both output synapses in thalamus and superior colliculus, uses different voltage gated Ca channels to mediate release at each synapse. This deviation from Dale’s law has been demonstrated at other synapses, but as far as I know, not here in the early visual system, where it is likely important for differential roles in calculations at distinct retinal output targets.

COMMENTS

This nicely performed study examines the differential mechanisms of synaptic release from presumed single retinal ganglion cell axons at two post-synaptic targets, the thalamus and the superior colliculus in adult mouse brain slices.

Careful and thorough voltage clamp analysis documents that SC synapses are highly dependent on N-type calcium channels, while synapses at a different target of the same axons, in the thalamus, utilize P/Q type calcium channels.

The results are convincing, and have presumed implications for visual function, to be addressed in future experiments.

I only have minor issues:

1) please define adult mouse. It’s probably better to just list the age range of animals.

2) .1% DMSO is actually a pretty high concentration. Any experiments using this concentration should use vehicle controls to assess any drug effects.

3) Line 181. The logic does not follow. Existence of L channels in soma/dendrites does not preclude an additional location in presynaptic terminals.

4) There is something wrong with the dnqx/cpp traces in Fig 1C, in which it appears that the stimulus artifact was reduced by these drugs, which is not expected.

5) Figures and legends should be consistent with text, and indicate extracellular Ca concentration as Ca[subscript]e

6) The discussion was interesting and provocative, but leaves the reader wanting a little bit more. Can the speculation be made a bit more specifically, regarding why the two synapses use different Ca channels? Is it for high frequency fidelity? If so, how might this be tested.

-

REVIEWER 2

ADVANCES IN THE FIELD

This is a straightforward study which explores the contribution of CaV2.2 and CaV2.1 channels (N-type and P-type currents) to synaptic transmission between retinal ganglion cell projections and postsynaptic neurons in dorsal lateral geniculate nucleus (dLGN) and superior colliculus (SC). The use of highly selective toxin inhibitors of these channels show that synaptic transmission to SC neurons is primarily inhibited by w-CgTxGVIA and to dLGN by w-AgaIVA. These findings should be interesting to the retina field and more generally represents another intriguing example of presynaptic specificity based on whether CaV2.1 or CaV2.2 channels dominate in transmitter release.

This should be a tractable system to assess if there are properties unique to one or other channel, that endows unique features at these synapses.

Therefore, overall, the information is useful, and as far as I know , not previously reported.

The sweeping conclusions about single axons using different channels depending on target organ has not been demonstrated and the authors conclusions are too far reaching on this point.

STATISTICS

The number of recordings in the manuscript is small, the data are consistent and conclusions about the large changes are appropriate. But, for smaller effects, it is not appropriate to use a p value cut off to make binary statements about whether a given drug did or did not affect the synaptic response, given the same sample size relative to the variance across recordings.

COMMENTS

This is a straightforward study which explores the contribution of CaV2.2 and CaV2.1 channels (N-type and P-type currents) to synaptic transmission between retinal ganglion cell projections and postsynaptic neurons in dorsal lateral geniculate nucleus (dLGN) and superior colliculus (SC). The use of highly selective toxin inhibitors of these channels show that synaptic transmission to SC neurons is primarily inhibited by w-CgTxGVIA and to dLGN by w-AgaIVA. These findings should be interesting to the retina field and more generally represents another intriguing example of presynaptic specificity based on whether CaV2.1 or CaV2.2 channels dominate in transmitter release. This should be a tractable system to assess if there are properties unique to one or other channel, that endows unique features at these synapses.

A few comments/suggestions

1. Figure 1. It seems unfortunate to have captured beautiful images of biocytin filled neurons, but show some quantification across cells that captures the differences between them.

2. The authors suggest that single retinal ganglion cells use either CaV2.2 (N-type) or CaV2.1 (P-type) at presynaptic termini depending on the type of target neuron. The authors have not addressed this question and their conclusions are too far reaching on this point, absent additional data, and should be toned down.

a. Are there cell transcriptome or immuno data in the literature that the authors can quote, that show both Cacna1a and Cacna1b are expressed in relevant populations of retinal ganglion cells?

b. The authors highlight that “the great majority of RGC axons bifurcate and project to both dLGN and SC” (lines 34, 74, 233 in significance, intro and discussion). The population sample size in this study is small, between 5 and 7 recordings, so the authors need to calculate whether this small sample size is sufficient to assume that they have captured postsynaptic recordings that are representative of bifurcating axons.

3. What does “n” refer to, number of animals, number of drug applications, number of individual recordings, number of slices? This should be defined for each figure in figure legends.

4. The authors need to plot individual data points in Fig. 3. The data are obscured by collapsing into histograms.

5. Line 191-194. The authors conclude that “bath application of the N-type channel blocker CgTX (1μM) had little or no effect on EPSC amplitude, but the subsequent application of the P/Q-type blocker AgTX (0.2-0.4 μM) led to a substantial reduction. By contrast in SC, EPSC amplitude was greatly affected by N-type, but not by P/Q-type blockade.” The summary that N-type don’t contribute to dLGN synapses and P/Q do not contribute to SC synapses ignores the average 14-18% block of the less effective toxin in each case. (e.g. “One-way ANOVA with Tukey test p < 0.001) but not after P/Q blockade (AgTX, n=5, 17.5 {plus minus} 6.9%, p > 0.2).”) Even though difference did not reach significance as defined by the p values, based on the variability across recordings, w-AgaIVA must have inhibited some synapses. Showing individual data points would be preferable so that the reader can assess the variability variance associated with each blocker/preparation. So, for smaller effects, it is not appropriate to use a p value cut off to make binary statements about whether a given drug did or did not affect the synaptic response, given the same sample size relative to the variance across recordings.

6. Use full names of toxins w-CgTx GVIA and w-AgaIVA throughout manuscript, there are other conus and aga toxins that have different specificities

Minor

1. Add a comment to explain the residual signal in Fig. 1 for SC neurons in panel C.

2. Line 159-161 “indicating that the amplitude of the initial EPSC was about 1.4-1.7 fold larger than the second one (Fig. 1C). This should be smaller, not larger?

3. Line 117 in methods - w CgTx GVIA is referred to as GIVA

4. Suggest using [Ca]ex or something more standard than Cae

5. Line 232. The use of “asymmetric arrangement” doesn’t seem the right descriptor perhaps “ use of different presynaptic CaV channels depending on postsynaptic target neuron"

6. There are several reports of either N-type or P-type currents dominating at specific synapses - could also add https://doi.org/10.1016/S0896-6273(00)81246-5
